# [^177^Lu]Lu-PSMA-617 in Metastatic Hormone-Sensitive Prostate Cancer: Evidence from PSMAddition and Considerations for Treatment Sequencing

**DOI:** 10.3390/curroncol33100624

**Published:** 2026-10-16

**Authors:** Carlo Buonerba, Giuseppe Di Lorenzo

**Affiliations:** 1 Associazione O.R.A. ETS—Oncology Research Assistance, 84134 Salerno, Italy; 2 School of Medicine, UniCamillus—Saint Camillus International University of Health Sciences, 00131 Rome, Italy

**Keywords:** [^177^Lu]Lu-PSMA-617, metastatic hormone-sensitive prostate cancer, PSMAddition, prostate-specific membrane antigen, PSMA PET, androgen receptor pathway inhibitor, radioligand therapy, treatment sequencing

## Abstract

Introducing [^177^Lu]Lu-PSMA-617 into metastatic hormone-sensitive prostate cancer (mHSPC) raises questions about patient selection and treatment sequencing. This non-systematic narrative review examines PSMAddition alongside evidence on alternative treatments, prostate-specific membrane antigen (PSMA) imaging, safety, and subsequent therapy. In the peer-reviewed analysis, with a data cutoff of 13 January 2025, adding radioligand therapy to androgen-deprivation therapy and an investigator-selected androgen receptor pathway inhibitor improved radiographic progression-free survival (hazard ratio 0.72; 95% confidence interval 0.58–0.90). Overall survival was immature and did not meet the prespecified interim significance threshold (hazard ratio 0.84; 95% confidence interval 0.63–1.13). A subsequent non-peer-reviewed sponsor update supported the radiographic findings, although the updated analysis was not controlled for type I error and overall survival remained immature. Radioligand intensification increased grade ≥3 adverse events (51% versus 43%). Prespecified composite quality-of-life and pain endpoints showed no statistically significant differences, although their interpretation is limited by the inclusion of progression or death. Control-arm crossover after progression was optional. Direct mHSPC evidence is distinguished from contextual castration-resistant evidence. Neither a preferred hormonal partner nor superiority over docetaxel-containing triplet therapy has been established. A non-prescriptive five-domain framework integrates target expression, disease characteristics, patient fitness, subsequent treatment options, and delivery capacity to support multidisciplinary assessment.

## Simple Summary

Radioligand therapy delivers radiation to prostate cancer cells that express a protein called prostate-specific membrane antigen (PSMA). PSMAddition tested adding this treatment to hormonal therapy earlier in patients whose cancer had spread but still responded to hormone treatment. Published results showed that it delayed worsening on scans or death, but increased side effects, including severe adverse events and dry mouth. Longer follow-up is needed to determine whether patients live longer. Quality-of-life and pain worsening endpoints did not differ significantly between groups; this does not establish equivalent wellbeing or absence of harm. Some patients initially assigned to hormonal therapy alone received radioligand therapy after progression, but later treatment was not guaranteed. This review considers the trial alongside other treatments, scan findings, patients’ general health, future treatment options, and access to specialist services. It offers a framework to help clinical teams and patients discuss these choices.

## 1. Introduction

Adding [^177^Lu]Lu-PSMA-617 to established hormonal treatment raises a practical question: which patients should receive radioligand therapy early, and how should it fit with other effective treatments? On 31 July 2026, the US Food and Drug Administration approved [^177^Lu]Lu-PSMA-617 with an androgen receptor pathway inhibitor (ARPI) for adults with prostate-specific membrane antigen (PSMA)-positive metastatic androgen pathway modulation-naive or -sensitive prostate cancer (mAPMN/S) [1]. The trial report uses androgen pathway modulator-naive/sensitive terminology [2]. This review uses the familiar terms metastatic hormone-sensitive prostate cancer (mHSPC) and metastatic castration-resistant prostate cancer (mCRPC), retaining source-specific terminology when describing the indication or publication title. Both PSMAddition groups received ongoing androgen-deprivation therapy (ADT) with a gonadotropin-releasing hormone agonist or antagonist or had undergone bilateral orchiectomy [2].

VISION established overall-survival and radiographic progression-free survival benefits after ARPI and taxane therapy in mCRPC [3], and PSMAfore extended the evidence to taxane-naive mCRPC [4]. TheraP compared [^177^Lu]Lu-PSMA-617 with cabazitaxel; ENZA-p evaluated its addition to enzalutamide using an adaptive treatment schedule; UpFrontPSMA assessed sequential radioligand therapy and docetaxel in de novo high-volume mHSPC; and BULLSEYE evaluated response-adapted radioligand therapy in oligometastatic hormone-sensitive disease [5,6,7,8].

In later mCRPC, treatment selection is strongly influenced by prior therapy, residual target expression, cumulative toxicity, and the immediate need for disease control. In mHSPC, patients may live for many years and have several effective intensification options. Earlier disease control must therefore be weighed against treatment tolerance, alternatives to radioligand therapy, cumulative organ exposure, and the treatments likely to be needed after progression.

PSMA positron-emission tomography (PET) is central to eligibility. A positive scan identifies disease that can be targeted, but it does not by itself determine expected absolute benefit or optimal timing. Uptake intensity, total PSMA-positive tumour burden, and clinically important target-poor lesions may inform assessment. Their role beyond the trial-defined eligibility criteria is discussed separately from the evidence for treatment efficacy.

### Narrative Review Approach

This clinically focused, non-systematic narrative review integrates published evidence with expert clinical interpretation. PubMed was the sole bibliographic database used to identify the journal literature. Selected conference abstracts and presentations, regulatory documents, prescribing information, and sponsor-reported updates were consulted as supplementary sources.

Evidence was selected according to its clinical relevance to PSMAddition, PSMA-targeted radioligand therapy, imaging-based selection, safety, dosimetry, and treatment sequencing. Priority was given to pivotal randomised trials and prospective studies in metastatic hormone-sensitive prostate cancer, while evidence from castration-resistant disease was used for contextual interpretation. The evidentiary status of peer-reviewed publications and preliminary or sponsor-reported findings was distinguished throughout.

Both authors conducted the literature search and contributed to the conceptualisation and review of the manuscript. No prespecified systematic search or formal study-selection protocol was used. Original PubMed search dates were not recorded; targeted verification of cited publications and supplementary sources was completed on 11 October 2026. The purposive approach may not capture all relevant evidence.

## 2. Clinical Evidence for [^177^Lu]Lu-PSMA-617 in Metastatic Hormone-Sensitive Prostate Cancer

### 2.1. PSMAddition Design and Efficacy

PSMAddition was an international, randomised, open-label phase III trial conducted at 169 sites in 20 countries. It randomly assigned 1144 men with PSMA-positive mHSPC; 50% had de novo metastatic disease and 68% had high-volume disease [2]. Patients were treatment-naive or minimally treated for metastatic disease; up to 45 days of ADT, an ARPI, or both before informed consent was permitted [2,9]. Both groups received ADT plus an investigator-selected ARPI, with or without [^177^Lu]Lu-PSMA-617 at 7.4 GBq (±10%) every six weeks for up to six administrations. Eligibility required metastatic disease on conventional imaging and at least one tumour lesion with centrally read [^68^Ga]Ga-PSMA-11 uptake greater than normal liver; patients were excluded if all present liver metastases were PSMA-negative [2,9]. Patients with metastases detected only on PSMA PET, but not on conventional imaging, were not represented; applicability to this population remains uncertain. The primary endpoint was centrally assessed radiographic progression-free survival, defined as progression by Prostate Cancer Clinical Trials Working Group 3-modified Response Evaluation Criteria in Solid Tumours (RECIST) version 1.1 or death [2]. The ADT–ARPI control allowed assessment of the additional effect of radioligand therapy.

The peer-reviewed analysis had a data cutoff of 13 January 2025 and a median interval from randomisation to cutoff of 23.6 months; median follow-up for radiographic progression-free survival was 19.6 months. It was the second interim analysis of radiographic progression-free survival and the first interim analysis of overall survival [2,10]. Radiographic progression or death occurred in 139 of 572 patients receiving radioligand intensification and 172 of 572 controls. The hazard ratio (HR) was 0.72 (95% confidence interval 0.58–0.90; one-sided *p* = 0.0021), with median radiographic progression-free survival unreached in either group [2]. Overall survival remained immature and did not meet the prespecified interim significance threshold (hazard ratio 0.84; 95% confidence interval 0.63–1.13; one-sided *p* = 0.125) [2,10].

A separate, non-peer-reviewed sponsor-reported analysis used a 17 March 2026 cutoff and median follow-up of 37.7 months. Described by the sponsor as the final radiographic progression-free survival analysis and second interim overall-survival analysis, it yielded hazard ratios of 0.67 (95% confidence interval 0.55–0.82) and 0.80 (0.63–1.01), respectively [11,12]. The updated radiographic analysis was not controlled for type I error; the overall-survival result remained interim and did not establish a survival benefit. This update has not been published in full, and the final overall-survival analysis remains pending [12].

Because median radiographic progression-free survival was unreached in both groups at the published cutoff, a difference between the medians could not be estimated. The crude proportions with radiographic progression or death were 24.3% (139/572) versus 30.1% (172/572), a difference of 5.8 percentage points. These are event proportions over the observed follow-up, not fixed-time Kaplan–Meier risks or a number needed to treat. The primary report plots the survival curves but does not tabulate exact landmark probabilities with confidence intervals; it reports 153 versus 134 patients at risk at 24 months [2]. Secondary findings at that cutoff included delayed prostate-specific antigen (PSA) progression (hazard ratio 0.42; 95% confidence interval 0.30–0.59) and delayed castration resistance (hazard ratio 0.70; 95% confidence interval 0.58–0.84) [2]. These findings support the radiographic result without establishing a survival benefit.

PSMAddition compared early radioligand intensification with ADT–ARPI and optional, protocol-permitted radioligand therapy after centrally confirmed radiographic progression, not mandatory delayed treatment. The intention-to-treat analysis estimates the effect of assignment to these strategies rather than the biological effect of immediate versus guaranteed delayed treatment. At the 13 January 2025 cutoff, 91/572 controls had crossed over (15.9% of the full control arm), corresponding to 91/152 patients with confirmed progression (59.9%) [2,10]. Non-peer-reviewed sponsor reporting gives a full-arm crossover proportion of 21% at the 17 March 2026 cutoff, without an updated post-progression denominator [12]. At the PSMAfore final overall-survival cutoff of 1 January 2025, 141/234 controls had crossed over (60.3%), corresponding to 141/187 patients with centrally confirmed radiographic progression (75.4%) [13]. These proportions should not be used to infer optimal sequencing across trials, given differences in follow-up, progression maturity, crossover eligibility, and access. Survival interpretation also depends on crossover timing, reasons for non-crossover, and subsequent treatment. Assumption-dependent crossover-adjusted analyses may complement, but cannot replace, the randomised intention-to-treat comparison.

### 2.2. Patient-Reported Outcomes, Safety, and Subgroup Analyses

The clinical value of delayed radiographic progression also depends on whether patients maintain function and quality of life and experience fewer symptoms. In the primary publication and a conference report of the prespecified secondary patient-reported outcome analysis, time-to-worsening endpoints combined clinically meaningful score deterioration, clinical progression, or death; non-composite time-to-worsening analyses excluding progression and death were post hoc [2,14]. Hazard ratios were 1.14 (95% confidence interval 0.98–1.33) for the Functional Assessment of Cancer Therapy-Prostate (FACT-P) total score, 1.13 (0.97–1.31) for the EuroQol 5-Dimension 5-Level (EQ-5D-5L) utility score, and 1.02 (0.87–1.18) for Brief Pain Inventory-Short Form (BPI-SF) pain intensity [2,14]. An HR above 1 indicates a higher hazard of composite worsening with radioligand intensification, not improved quality of life; all confidence intervals included unity. These results establish neither equivalence nor absence of harm. Prespecified longitudinal analyses were also reported: FACT-P scores showed greater deterioration during the radioligand treatment period, whereas EQ-5D-5L and pain profiles were generally similar between groups [2]. More mature longitudinal and component-level reporting is needed to separate treatment burden from later disease-control effects. Time to first symptomatic skeletal event or death also did not differ significantly (hazard ratio 0.89; 95% confidence interval 0.62–1.26), with medians unreached in both groups [2,14].

At the 13 January 2025 cutoff, safety was analysed in treated participants according to treatment received. All-cause grade 3 or worse adverse events occurred in 286 of 564 patients (51%) receiving radioligand intensification and 243 of 565 controls (43%) [2]. Serious adverse events of any cause occurred in 180 patients (32%) and 162 patients (29%), respectively; 17 patients (3%) in the radioligand arm had a serious event attributed to [^177^Lu]Lu-PSMA-617 [2]. Dry mouth was the most common adverse event (46% vs. 4%); all cases were grade 1 or 2 and none was serious. Grade 3 or worse cytopenias occurred in 14.4% versus 5.0%, and adverse events led to discontinuation of any study treatment in 16.1% versus 9.0% [2,10]. These findings show increased severe adverse events, cytopenias, and treatment discontinuation with radioligand intensification. Longer observation is needed to assess delayed effects in patients treated earlier in their disease course.

Conference-reported subgroup analyses showed broadly similar radiographic progression-free survival hazard ratios across disease-volume and de novo versus recurrent metastatic categories [15]. Subgroup confidence intervals were wider, and a non-significant interaction does not establish equal benefit. Disease volume and metastatic presentation may influence prognosis, expected absolute benefit, and clinical urgency, but neither has been validated as a predictive marker for selecting early radioligand therapy over other intensification strategies or for safely deferring it.

### 2.3. Context from Other Prospective Radioligand Trials

Earlier trials clarify the context of PSMAddition. VISION demonstrated a survival benefit in heavily pretreated PSMA-positive mCRPC, while PSMAfore improved radiographic progression-free survival over an ARPI change before taxane chemotherapy [3,4]. In the final PSMAfore analysis, median overall survival was 24.48 versus 23.13 months (intention-to-treat hazard ratio 0.91; 95% confidence interval 0.72–1.14) [13]. An inverse-probability-of-censoring analysis estimated a hazard ratio of 0.59 (0.38–0.91) [13]. The difference between these estimates illustrates the importance of the crossover exposure described above and the assumptions required for adjustment.

PSMAfore also reported favourable patient-reported outcomes compared with an ARPI change [16]. Its contrast with PSMAddition may reflect differences in disease state, comparator, baseline symptoms, endpoint construction, or follow-up; it does not establish a uniform patient-reported effect across settings.

ENZA-p evaluated first-line enzalutamide with or without adaptively administered [^177^Lu]Lu-PSMA-617 in 162 men with high-risk mCRPC. Interim PSMA PET determined whether patients received two or four administrations. Median PSA progression-free survival was 13.0 versus 7.8 months (hazard ratio 0.43; 95% confidence interval 0.29–0.63); later follow-up reported median overall survival of 34 versus 26 months (hazard ratio 0.55; 0.36–0.84) and benefits in selected quality-of-life domains [6,17]. Thirty of 79 controls (38%) subsequently received [^177^Lu]Lu-PSMA-617 outside the trial [17]. The observed survival benefit supports combination treatment in this selected mCRPC population.

Three phase II studies provide direct evidence in specific hormone-sensitive settings. UpFrontPSMA tested two 7.5-GBq administrations of [^177^Lu]Lu-PSMA-617 followed by six docetaxel cycles; both groups received ADT and neither received an ARPI. Undetectable PSA at 48 weeks occurred in 41% versus 16% (odds ratio 3.88; 95% confidence interval 1.61–9.38), with no demonstrated overall-survival difference at the available follow-up (hazard ratio 0.83; 0.38–1.83) [7]. BULLSEYE enrolled patients with recurrent PSMA-expressing oligometastatic hormone-sensitive disease and a PSA doubling time below six months. Per-protocol median progression-free survival was 25 versus 5 months (hazard ratio 0.07; 95% confidence interval 0.03–0.17) [8]. The comparator was active monitoring with deferred ADT rather than active systemic treatment. Both that comparator and the per-protocol analysis limit transfer of the estimate to patients receiving contemporary systemic intensification.

CONSOLIDATE evaluated two 7.4-GBq administrations of [^177^Lu]Lu-PSMA-617 as consolidation for residual PSMA-positive disease after androgen deprivation and docetaxel in 30 patients with synchronous high-volume mHSPC. A prostate-specific antigen concentration of 0.2 ng/mL or lower at six months occurred in 60% versus 13% with protocol-permitted standard care (risk ratio 4.5; 95% confidence interval 1.2–17.4), and no grade 3–4 toxicity was observed [18]. The trial was closed early because of poor accrual and changing treatment standards; its small sample, biochemical primary endpoint, and post-docetaxel consolidation design address a different sequencing question from PSMAddition.

The principal prospective radioligand trials are summarised in Table 1.

The studies differ in population, comparator, imaging criteria, and treatment schedule. These differences determine how far their findings can inform the hormone-sensitive setting.

## 3. Positioning Within Contemporary Treatment Strategies for mHSPC

### 3.1. Choice of Androgen Receptor Pathway Inhibitor

In PSMAddition, investigators selected the ARPI according to local authorisation [1,2]. In the investigator presentation’s treated-population table (*n* = 1129, rather than the 1144 randomised participants), the first assigned ARPI was most often abiraterone (37.6%), followed by apalutamide (32.9%) and enzalutamide (22.1%) [10]. Darolutamide and other ARPIs were less frequent. The trial was not designed or powered to compare partners. It therefore supports radioligand intensification of ADT plus an investigator-selected ARPI, rather than a uniquely validated combination with enzalutamide or another individual agent.

The percentages therefore describe initial ARPI assignment in the treated population, not the intention-to-treat efficacy population. The primary report permitted one ARPI switch without progression; this occurred in 52/564 patients (9.2%) in the radioligand safety group and 58/565 (10.3%) in the control safety group [2]. Such ARPI switching is distinct from protocol-permitted radioligand crossover after progression. Neither changes the original randomised assignment used for intention-to-treat efficacy analysis.

ENZA-p supports the combination of enzalutamide and [^177^Lu]Lu-PSMA-617 in its studied population [6,17]. Whether a particular ARPI changes the radioligand treatment effect in mHSPC remains unknown. Disease state, patient selection, and the adaptive ENZA-p schedule differ from PSMAddition, and the schedule should not replace the phase III regimen outside a trial.

ARPI choice also depends on prior exposure, cardiovascular and metabolic risk, falls or seizures, drug interactions, glucocorticoid requirements, cognition, adherence, cost, and availability. In a retrospective cohort of 256 previously ARPI-exposed patients with mCRPC, concurrent ARPI use during [^177^Lu]Lu-PSMA-617 was not associated with better adjusted overall survival (hazard ratio 1.03; 95% confidence interval 0.68–1.55) or a higher probability of a PSA decline of at least 50% (PSA50) [20]. Its imbalanced groups and different disease setting prevent conclusions about ARPI selection in mHSPC.

### 3.2. Relationship to Established Doublet and Triplet Strategies

Treatment intensification is established in mHSPC. Randomised trials have demonstrated improved outcomes with enzalutamide, apalutamide, abiraterone, or darolutamide added to androgen-deprivation therapy [21,22,23,24,25]. Docetaxel improved survival in appropriately selected patients, with a particularly clear early signal in high-volume disease [26]. ARASENS and PEACE-1 subsequently established survival benefits for docetaxel-containing triplet strategies in relevant populations [27,28]. A living network meta-analysis supports the general value of intensification while also showing that treatment effects and certainty vary by regimen and clinical subgroup [29].

PSMAddition did not directly compare radioligand intensification with docetaxel-containing triplet therapy or test its added value separately for each ARPI doublet. This matters for external applicability: 68% of participants had high-volume disease and 50% had de novo metastases [2], and similar fit patients may be considered for a docetaxel-containing triplet [27,28]. The trial establishes a benefit over an ADT–ARPI backbone, not a relative benefit versus an established triplet. Differences in population, imaging selection, endpoints, follow-up, and later treatment preclude ranking these strategies by comparing hazard ratios across trials.

Treatment selection therefore remains individualised. Disease tempo, symptoms, distribution, chemotherapy fitness, expected toxicity, patient preference, and access all matter. Docetaxel-containing therapy is an established option for fit patients; radioligand intensification adds an option when target expression, organ function, and delivery capacity support its use. These considerations guide discussion but are not validated predictors of differential radioligand benefit.

Although the radioligand course is finite, patients continue ADT and an ARPI [9]. Its practical burden therefore comprises additional visits, laboratory monitoring, adverse effects, and radiation precautions rather than replacement of daily hormonal treatment. These requirements should be discussed alongside the burdens of chemotherapy and hormonal treatment alone.

Table 2 summarises the principal clinical and practical distinctions without ranking the regimens.

## 4. PSMA PET Selection Criteria and Imaging Considerations

### 4.1. Trial-Specific PSMA PET Criteria

PSMA PET has changed the staging of high-risk and metastatic prostate cancer and provides molecular target assessment for radioligand therapy. In proPSMA, PSMA PET/computed tomography (PET/CT) was more accurate than conventional imaging for initial staging of high-risk disease [30]. For treatment selection, the relevant question extends beyond detection to whether the disease most likely to determine prognosis or symptoms expresses sufficient target for effective radiation delivery.

The major radioligand trials used different definitions of PSMA-positive disease. VISION required at least one lesion with uptake visually greater than liver and excluded qualifying PSMA-negative lesions [3]. TheraP used higher quantitative thresholds and fluorodeoxyglucose (FDG) PET to exclude metabolically active PSMA-negative disease [5,31]. PSMAfore used liver-referenced central assessment with exclusionary-lesion rules [4]. ENZA-p and UpFrontPSMA applied higher uptake thresholds; UpFrontPSMA also excluded major FDG discordance [6,7]. PSMAddition required conventional-imaging metastases and at least one tumour lesion with [^68^Ga]Ga-PSMA-11 uptake above normal liver, excluding patients if all present liver metastases were PSMA-negative; FDG PET was not required [2,9]. Of 1420 patients who underwent PSMA PET/CT, 1232 (86.8%) were centrally classified as positive [2,32]. Table 3 compares these eligibility criteria and identifies a reporting difference between the ENZA-p primary and biomarker publications. PSMAddition’s requirement for conventional-imaging metastases limits extrapolation to PSMA PET-only disease. Liver-referenced visual thresholds should not automatically be assumed equivalent across PSMA PET tracers, because biodistribution and hepatic background activity differ [33].

### 4.2. Uptake, Tumour Volume, and Heterogeneity

A positive scan can represent markedly different disease patterns. Uptake intensity may influence absorbed tumour dose, but varies across lesions and with scanner calibration, reconstruction, timing, tracer, and partial-volume effects. Total PSMA-positive tumour volume distinguishes a few intensely avid lesions from extensive target-expressing disease. Lesion-level heterogeneity can identify sites likely to receive less radiation despite an overall positive scan.

Biomarker analyses in mCRPC examine these features. In the prespecified TheraP analysis, the odds ratio for a prostate-specific antigen decline of at least 50% with [^177^Lu]Lu-PSMA-617 versus cabazitaxel was 12.19 (95% confidence interval 3.42–58.76) among patients with baseline PSMA mean standardised uptake value (SUV_mean_) of at least 10 and 2.22 (1.11–4.51) below that cut point (adjusted interaction *p* = 0.039); high FDG metabolic tumour volume was associated with a lower response irrespective of assigned treatment [31]. In the prespecified ENZA-p substudy, baseline PSMA total tumour volume was prognostic for overall survival and modified the overall-survival treatment effect (interaction *p* = 0.0078), whereas SUV_mean_ did not (*p* = 0.88) [34]. In the exploratory VISION analysis, whole-body tumour SUV_mean_ was the quantitative PET variable most strongly associated with treatment efficacy. Treatment-effect estimates favoured radioligand therapy across SUV_mean_ quartiles, but the confidence intervals included unity in the lowest quartile for both radiographic progression-free and overall survival [35]. The analyses differ in endpoints, comparators, and statistical methods; they should not be interpreted as interchangeable biomarker validations.

The distinction between prognostic and predictive information is relevant to interpretation. A marker may identify patients with a worse expected outcome regardless of treatment without demonstrating that radioligand therapy produces a larger relative benefit. Conversely, a treatment interaction can be suggested in an exploratory analysis but still require prospective validation. No quantitative PSMA PET threshold, tumour-volume cut point, or heterogeneity metric has been validated to add to the PSMAddition eligibility criterion in routine mHSPC practice.

### 4.3. FDG Discordance and Anatomical Risk

TheraP excluded FDG-positive/PSMA-negative disease because metabolically active target-poor lesions may receive inadequate PSMA-directed radiation [5,31]. The prevalence and importance of discordance may differ in mHSPC. Requiring FDG PET routinely would change access, cost, and the population represented by PSMAddition, without direct evidence that this improves treatment selection in that setting.

Anatomical context may be as important as whole-body averages. A patient can satisfy trial eligibility while harbouring a target-poor liver lesion, epidural mass, bulky nodal deposit, or other site that carries disproportionate clinical risk. Such findings may warrant biopsy, local treatment, an alternative systemic strategy, or closer surveillance even when radioligand treatment remains appropriate for the remainder of the disease. The clinically relevant imaging assessment therefore includes the distribution of target expression and the relationship between target-poor lesions and symptoms or organ threat.

In practice, PSMA PET should be interpreted against the applicable indication and product information [9]. Additional quantitative or dual-tracer findings may inform multidisciplinary assessment. Harmonised acquisition, reading, segmentation, and reporting remain important.

## 5. Clinical Assessment, Treatment Sequencing, and Implementation

### 5.1. Patient Characteristics and Safety Considerations

The joint European Association of Nuclear Medicine/Society of Nuclear Medicine and Molecular Imaging (EANM/SNMMI) procedure guideline and the SNMMI consensus statement recommend assessment of blood counts, renal function, performance status, disease distribution, prior therapy, and radiation safety [36,37]. Developed largely in advanced disease, they remain useful for baseline evaluation and monitoring, while longer-term safety after treatment in mHSPC requires separate follow-up.

Marrow assessment includes baseline anaemia or thrombocytopenia, the extent of bone involvement, prior pelvic radiotherapy, and previous marrow-toxic treatment. A retrospective mCRPC cohort associated clinically significant haematologic toxicity with pre-existing cytopenia, high bone tumour burden, and prior taxane exposure [38]. For patients treated earlier, anticipated future chemotherapy and recovery between cycles also matter; a single laboratory threshold cannot capture all of these risks.

Renal uptake and excretion make kidney function another routine consideration. Severe nephrotoxicity has been uncommon in trial and dosimetry studies of the standard [^177^Lu]Lu-PSMA-617 course, and a small retrospective series of selected patients with baseline impairment reported generally stable renal function [39,40]. These observations are reassuring but derive from limited follow-up and selected advanced-disease populations; they do not establish the lifetime renal consequences of treatment during mHSPC. Hydration, avoidance of preventable nephrotoxins, serial renal assessment, and review of obstructive uropathy remain appropriate [9].

Salivary and lacrimal exposure can produce xerostomia, taste change, or ocular symptoms. These effects are often lower grade but may be important when treatment is offered to patients with few cancer-related symptoms. Baseline oral health, pre-existing sicca symptoms, and the patient’s tolerance for possible chronic dryness are relevant to counselling. Evidence for specific protective interventions remains limited, and unproven approaches should not replace dose, schedule, and toxicity management consistent with product information and specialist guidance [9,36].

Dosimetry shows substantial variation in absorbed dose between patients and lesions [41,42]. Administered activity (GBq) is not the same as absorbed dose (Gy): identical activities can produce different tumour and organ exposures. PSMAddition evaluated 7.4 GBq every six weeks for up to six administrations; its published selection and prescription procedures do not describe an absorbed-dose eligibility threshold or a dosimetry-guided activity algorithm [2]. The July 2026 US prescribing information specifies six doses, with discontinuation for progression or unacceptable toxicity and interruption, reduction, or discontinuation for adverse reactions [9].

Pretreatment PSMA PET assesses target expression and tumour distribution, whereas post-therapy quantitative single-photon emission computed tomography/computed tomography (SPECT/CT) measures the therapeutic radionuclide’s activity distribution and supports time-integrated activity and absorbed-dose estimation [43,44]. Reliable quantification requires camera calibration, validated reconstruction with attenuation and scatter correction, attention to partial-volume effects, reproducible segmentation and registration, and documented acquisition timing. Sampling and time–activity curve assumptions contribute uncertainty that should accompany dose estimates [43,44].

Cumulative marrow and kidney exposure is relevant to haematologic reserve, renal function, and later treatment; salivary- and lacrimal-gland exposure may contribute to persistent dryness [39,43]. Small-gland delineation and heterogeneous bone disease complicate dose estimation, and organ-average dose does not capture every local exposure. Earlier treatment and possible retreatment increase the importance of recording cumulative activity, organ-dose estimates when available, and long-term clinical outcomes. Harmonised dose measurements, validated tumour dose–response and organ dose–toxicity relationships, and prospective comparisons with the phase III regimen are needed before dosimetry-guided prescription or response-adapted administration can replace it in mHSPC [36,41,43,44].

### 5.2. Treatment Sequencing and Retreatment

Patients treated early may later need docetaxel, cabazitaxel, poly(ADP-ribose) polymerase (PARP) inhibition or another molecularly selected therapy, local treatment for dominant lesions, further radiopharmaceutical treatment, or an alpha-emitter trial. PSMAddition does not establish the order of these options. Interpretation of early treatment will also depend on which therapies patients receive after progression and how well they tolerate them.

The published delay in castration resistance provides supporting evidence for longer disease control [2], but radiographic progression-free survival does not describe the entire treatment course. Second progression-free survival (PFS2), measured from randomisation to investigator-assessed radiographic, clinical, or PSA progression on next-line therapy or death, was a prespecified PSMAddition secondary endpoint and was immature at the published cutoff [2]. Mature PFS2, subsequent-treatment exposure and tolerance, cumulative toxicity, symptom control, quality-adjusted survival, and treatment burden across successive lines are needed to assess outcomes beyond first progression; they cannot be inferred from the initial radiographic benefit alone.

Retreatment has been reported in selected mCRPC patients who responded initially and later progressed [45]. The principal report followed a prospective phase II cohort, but retreatment itself was not randomised. Patients generally had preserved organ function and renewed PSMA-avid disease. These observations do not establish routine reuse after treatment in mHSPC. Prospective studies should address the interval between courses, imaging criteria, cumulative activity, organ doses, and alternative treatment.

Delayed haematologic effects warrant surveillance. A hypothesis-generating single-institution series identified therapy-related myeloid neoplasms in 5 of 381 patients (1.3%) treated with [^177^Lu]Lu-PSMA, at a median of 33.6 months after the first administration [46]. Advanced disease, prior chemotherapy or radiotherapy, cumulative exposure, clonal haematopoiesis, patient selection, competing mortality, and retrospective ascertainment prevent interpretation of this proportion as a radioligand-attributable incidence or an mHSPC risk estimate. The later non-peer-reviewed sponsor efficacy update does not by itself establish equally mature ascertainment of rare delayed haematologic events [12]. Long-term trial and registry follow-up remains necessary.

### 5.3. A Multidimensional Framework for Clinical Assessment

Clinical assessment can be organised across five domains: target, disease, patient, sequence, and system (Figure 1). The five domains are an author-proposed conceptual synthesis of the reviewed literature and expert clinical interpretation, not a formal consensus product. The framework is a non-validated discussion aid, not a score, treatment-selection algorithm, or source of eligibility restrictions beyond the indication and trial evidence. Table 4 maps the domains to clinical questions, supporting evidence, its directness and limitations, and multidisciplinary considerations; these descriptive assessments are not formal certainty grades.

Procedure guidance and consensus recommendations chiefly address eligibility, imaging, organ function, administration, and monitoring, largely in advanced mCRPC [36,37]. The framework complements them by focusing on a question raised by earlier use: should this eligible patient receive radioligand therapy now? It brings disease urgency, competing intensification, later treatment options, and delivery capacity into the same assessment.

The target domain considers whether the disease most likely to determine prognosis, symptoms, or organ threat is broadly PSMA-accessible. It includes overall distribution, uptake, volume, heterogeneity, and the presence of clinically important target-poor lesions. The disease domain considers metastatic burden, tempo, symptoms, visceral involvement, anatomical risk, de novo versus recurrent presentation, and the anticipated absolute benefit from additional disease control. These two domains distinguish technical eligibility from the clinical need for early intensification.

The patient domain considers performance status, marrow and renal reserve, baseline symptoms, comorbidity, life expectancy, concurrent medication, prior treatment, and preferences regarding toxicity and mode of delivery. The sequence domain examines the likely need for chemotherapy, molecularly targeted agents, local therapy, later radioligand treatment, and clinical trials. It also considers whether early radioligand treatment could affect access to, or tolerance of, later therapy.

The system domain asks whether the planned treatment and monitoring can be delivered on time, and whether waiting periods or travel requirements change its feasibility for this patient. Delivery feasibility is distinct from clinical appropriateness: isotope supply, treatment capacity, and travel barriers are not biological eligibility criteria. These constraints may worsen geographic and socioeconomic inequities in access.

The domains are considered together, without a required sequence. The framework complements product information, multidisciplinary review, and patient preference; it is not a score or eligibility algorithm (PSMA—prostate-specific membrane antigen).

### 5.4. Implementation, Treatment Burden, and Patient-Reported Outcomes

Delivery requires coordination between medical oncology, nuclear medicine, radiology, urology, pharmacy, radiation safety, and supportive care. Responsibilities should be clear for imaging review, scheduling, laboratory assessment, adverse-event management, and progression. Expansion into mHSPC increases the importance of planning these services before referrals increase.

Isotope supply, scanners, licensed treatment facilities, trained staff, and follow-up capacity vary between health systems, as do authorisation and reimbursement. Concentration in specialist centres can add travel, accommodation, and caregiver burdens. Waiting times, completion rates, and reasons for non-initiation after referral should therefore be documented alongside clinical outcomes.

Patient-reported evaluation should extend beyond time to first worsening. VISION showed delayed deterioration in several quality-of-life and pain outcomes in advanced mCRPC [47], but differs from PSMAddition in disease state, comparator, baseline symptoms, endpoints, and follow-up. In mHSPC, repeated assessments should capture function, salivary symptoms, fatigue, work and caregiver burden, and quality of life across later treatments.

Counselling with the nuclear medicine team should clarify the added treatment burden and confirm that hormonal therapy continues. Patients may weigh avoidance of particular chemotherapy toxicities against travel, radiation precautions, or caregiving constraints. Recording their priorities and the alternatives discussed makes the decision understandable across the treating team.

### 5.5. Evidence Gaps and Research Priorities

Mature survival and prespecified PFS2 results should be accompanied by complete crossover and subsequent-treatment reporting, with assumption-explicit sensitivity analyses and outcomes across the full treatment sequence. Longer-term patient-reported data are needed to determine whether disease control preserves function and quality of life across successive treatments.

Imaging priorities include prospective evaluation of PSMA total tumour volume, uptake heterogeneity, target-poor high-risk lesions, and selected FDG discordance using harmonised methods and treatment-interaction analyses. Prospective comparisons of the tested fixed-activity regimen with response-adapted or dosimetry-guided schedules, including retreatment, should incorporate harmonised absorbed-dose measurements, dose–response and dose–toxicity relationships, cumulative organ exposure, and long-term surveillance.

Comparative and sequencing trials should evaluate radioligand intensification against docetaxel-containing strategies, including toxicity, patient preference, cost, and later therapy. ARPI-specific interactions require prespecified analyses or dedicated randomisation. Registries should document delivery, access, long-term marrow and renal outcomes, retreatment, and rare delayed events.

## 6. Conclusions

PSMAddition establishes that adding [^177^Lu]Lu-PSMA-617 to ADT and an investigator-selected ARPI improves radiographic progression-free survival in trial-eligible PSMA-positive mHSPC, with increased toxicity. Overall survival remains immature and was not significantly improved at the peer-reviewed and subsequent sponsor-reported interim analyses. The effect on outcomes across the full treatment sequence—including subsequent therapy, cumulative toxicity, quality-adjusted survival, and long-term treatment burden—remains unresolved. Optional crossover does not equate to guaranteed delayed treatment.

Clinical decisions should integrate target expression, disease characteristics, patient fitness and preferences, alternative treatments, and delivery feasibility. Mature follow-up and comparative sequencing evidence are needed; the author-proposed five-domain framework provides a non-validated aid to multidisciplinary discussion, not an eligibility algorithm.

## Figures and Tables

**Figure 1:**
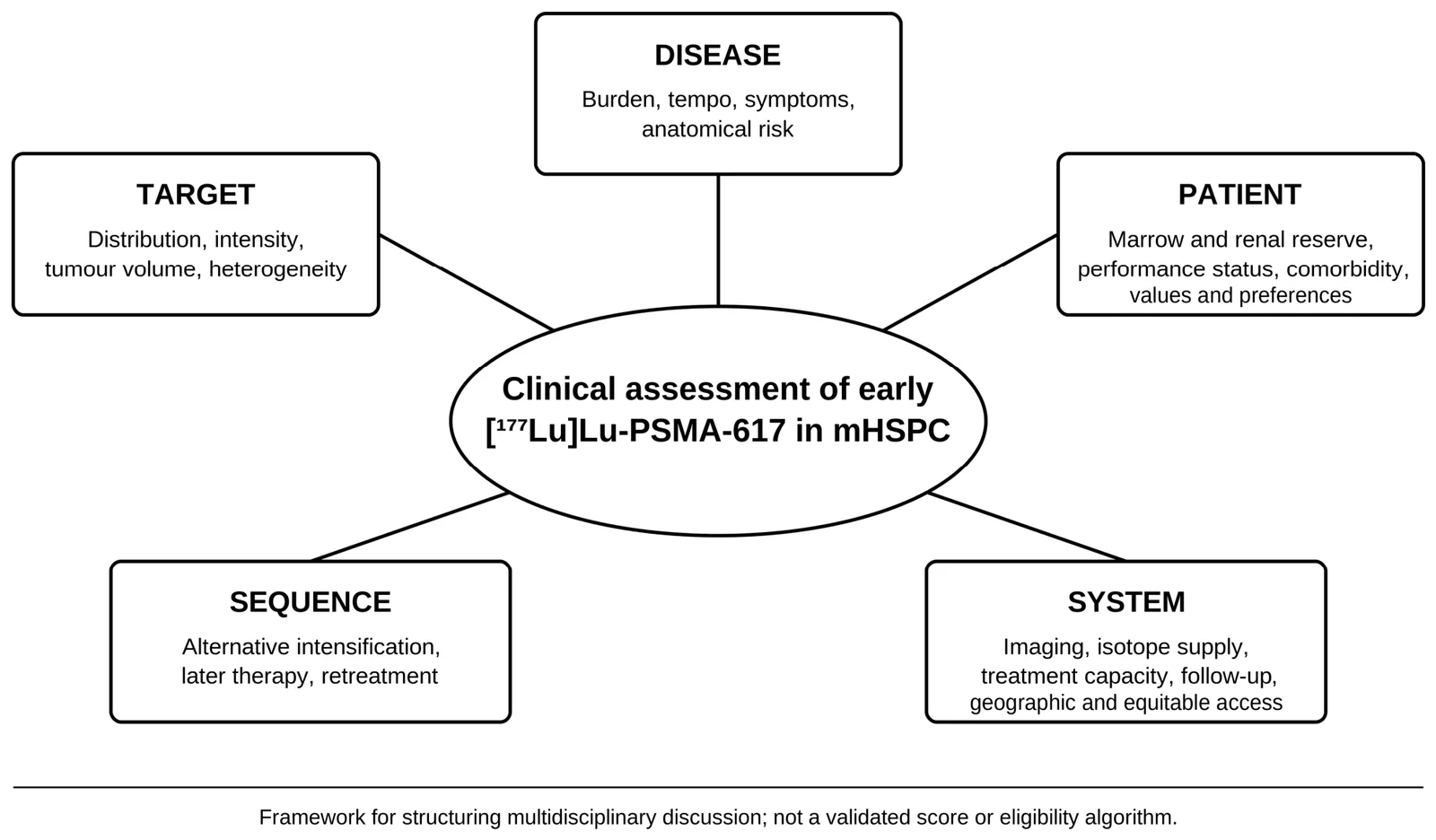
Author-proposed, non-validated five-domain framework for multidisciplinary assessment of early [^177^Lu]Lu-PSMA-617 in metastatic hormone-sensitive prostate cancer (mHSPC).

**Table 1:** Selected prospective trials informing the development of [^177^Lu]Lu-PSMA-617 across prostate cancer disease states.

Trial, Setting, and Analysis Context	Design and Radioligand Schedule	Principal Result	Relevance to mHSPC
VISION; post-ARPI, post-taxane mCRPC [3] *n* = 831 (OS, ITT); *n* = 581 (rPFS analysis set); median follow-up 20.9 months.	Phase III; [^177^Lu]Lu-PSMA-617 plus permitted standard care versus standard care; 7.4 GBq every 6 weeks for up to 6 cycles	rPFS HR 0.40 (99.2% CI 0.29–0.57); OS HR 0.62 (95% CI 0.52–0.74)	Established late-line efficacy; heavily pretreated mCRPC limits direct transfer
TheraP; post-docetaxel mCRPC [5,19] *n* = 200; ITT; median follow-up 35.7 months for the OS report.	Phase II; [^177^Lu]Lu-PSMA-617 versus cabazitaxel; 8.5 GBq initially, reduced by 0.5 GBq per cycle, up to 6 cycles	PSA50: 66% versus 37% (difference 29 percentage points; 95% CI 16–42); no statistically significant OS difference detected (36-month RMST difference −0.5 months; 95% CI −3.7 to 2.7)	Comparative activity under stringent dual-tracer enrichment; different disease state and comparator
PSMAfore; taxane-naive mCRPC after one ARPI [4,13,16] *n* = 468; ITT; median time from randomisation 24.11 months (updated rPFS) and 34.27 months (final OS).	Phase III; [^177^Lu]Lu-PSMA-617 versus ARPI change; crossover permitted; 7.4 GBq every 6 weeks for up to 6 cycles	Updated exploratory rPFS analysis: HR 0.49 (95% CI 0.39–0.61); final ITT OS HR 0.91 (0.72–1.14); 60.3% control crossover	Radioligand treatment versus ARPI change with optional post-progression crossover; disease remained castration-resistant
ENZA-p; first-line high-risk mCRPC [6,17] *n* = 162; ITT; median follow-up 20 months (primary PSA-PFS) and 34 months (OS).	Phase II; enzalutamide ± adaptive [^177^Lu]Lu-PSMA-617; 7.5 GBq every 6–8 weeks for 2 or 4 administrations	PSA-PFS HR 0.43 (95% CI 0.29–0.63); OS HR 0.55 (0.36–0.84)	Provides evidence of ARPI–radioligand complementarity; the adaptive schedule and mCRPC setting limit direct transfer
UpFrontPSMA; de novo high-volume mHSPC [7] *n* = 130 randomised; primary endpoint evaluable in 122 (61 per arm); median follow-up 2.5 years.	Phase II; 2 radioligand administrations followed by docetaxel versus docetaxel; ADT in both groups, no ARPI	Undetectable PSA at week 48: 41% versus 16% (OR 3.88; 95% CI 1.61–9.38); OS HR 0.83 (0.38–1.83)	Direct early-setting evidence within a sequential chemotherapy strategy
BULLSEYE; oligometastatic HSPC [8] *n* = 58 randomised; per-protocol efficacy analysis; median follow-up 27 months.	Phase II; [^177^Lu]Lu-PSMA-617 versus active monitoring with deferred ADT; 2 cycles, with 2 additional response-adapted cycles	Per-protocol median PFS 25 versus 5 months (HR 0.07; 95% CI 0.03–0.17)	The recurrent oligometastatic population, active-monitoring comparator, and per-protocol analysis limit generalisability to broader mHSPC
CONSOLIDATE; residual disease after ADT plus docetaxel [18] *n* = 30 randomised; primary biochemical assessment at 6 months.	Randomised phase II; 2 × 7.4 GBq every 6 weeks versus protocol-permitted standard care; *n* = 30	PSA ≤ 0.2 ng/mL at 6 months: 60% versus 13% (RR 4.5; 95% CI 1.2–17.4); no grade 3–4 toxicity	Small, early-terminated consolidation study with a biochemical primary endpoint
PSMAddition; PSMA-positive mHSPC [2,11,12,14] *n* = 1144 (ITT); *n* = 1129 (safety). Published cutoff: median 23.6 months from randomisation; rPFS follow-up 19.6 months.	Phase III; ADT plus investigator-selected ARPI ± [^177^Lu]Lu-PSMA-617; 7.4 GBq every 6 weeks for up to 6 cycles; crossover permitted	Peer-reviewed analysis, cutoff 13 January 2025: rPFS HR 0.72 (95% CI 0.58–0.90); interim OS HR 0.84 (0.63–1.13), not significant; 15.9% full-arm crossover Separate non-peer-reviewed sponsor update, cutoff 17 March 2026: rPFS HR 0.67 (0.55–0.82), not controlled for type I error; second interim OS HR 0.80 (0.63–1.01), survival benefit unestablished; 21% full-arm crossover; median follow-up 37.7 months	Principal phase III evidence; long-term sequencing and direct comparisons remain unresolved

Abbreviations: ADT, androgen-deprivation therapy; ARPI, androgen receptor pathway inhibitor; CI, confidence interval; HR, hazard ratio; HSPC, hormone-sensitive prostate cancer; ITT, intention to treat; mCRPC, metastatic castration-resistant prostate cancer; mHSPC, metastatic hormone-sensitive prostate cancer; OR, odds ratio; OS, overall survival; PFS, progression-free survival; PSA, prostate-specific antigen; PSA50, PSA decline of at least 50%; PSA-PFS, PSA progression-free survival; PSMA, prostate-specific membrane antigen; RMST, restricted mean survival time; rPFS, radiographic progression-free survival; RR, risk ratio. All primary and final journal reports cited in this table are peer-reviewed; the March 2026 PSMAddition sponsor update is a separate, non-peer-reviewed source. Follow-up descriptors refer to the specified analysis and are not interchangeable; the 6-month CONSOLIDATE assessment is an endpoint time, not median follow-up. Differences in populations, comparators, endpoints, and follow-up preclude cross-trial efficacy ranking.

**Table 2:** Qualitative comparison of mHSPC intensification strategies.

Dimension	ADT–ARPI Doublet	Docetaxel-Containing Triplet	Radioligand Intensification
Evidence and population	Phase III evidence for individual ARPIs; outcomes and populations differ across trials [21,22,23,24,25].	Phase III OS benefit from adding an ARPI to ADT–docetaxel in the populations studied [27,28].	PSMAddition: phase III rPFS benefit over ADT–ARPI; OS remains interim [2].
Eligibility	mHSPC; ARPI choice informed by comorbidity, interactions, and patient preference.	mHSPC with fitness for docetaxel; consider disease presentation and burden.	PSMA-positive, conventionally metastatic disease and adequate organ function; trial-specific imaging selection [2,9].
Principal toxicities	ADT effects plus ARPI-specific cardiovascular, metabolic, fatigue, falls, and interaction risks.	Doublet toxicities plus docetaxel-related myelosuppression, infection risk, neuropathy, and fatigue.	Doublet toxicities plus cytopenias, dry mouth, fatigue, and radiation-related organ exposure [2,9].
Duration and imaging	Ongoing ADT and ARPI; no treatment-specific PSMA PET requirement.	Finite docetaxel course, usually six cycles; ongoing ADT and ARPI; no treatment-specific PSMA PET requirement.	Up to six 7.4-GBq administrations every six weeks; ADT–ARPI continues; PSMA PET required [2,9].
Delivery burden	Medication, laboratory and clinical monitoring; agent-specific precautions.	Infusion visits, laboratory monitoring, and chemotherapy supportive care.	Specialist nuclear medicine capacity, isotope supply, monitoring, travel, and radiation precautions.
Unresolved comparisons	Optimal selection and sequencing among available intensification options.	No direct randomised comparison with PSMAddition-type intensification.	No direct triplet comparison, preferred ARPI, or established lifetime sequencing advantage; long-term toxicity remains important.

This table is a clinical comparison, not an indirect efficacy ranking. Trial populations and comparators differ; OS evidence for one ARPI regimen should not be transferred to every doublet. ADT, androgen-deprivation therapy; ARPI, androgen receptor pathway inhibitor; mHSPC, metastatic hormone-sensitive prostate cancer; OS, overall survival; PET, positron-emission tomography; PSMA, prostate-specific membrane antigen; rPFS, radiographic progression-free survival.

**Table 3:** Selected trial-specific PSMA PET eligibility criteria and interpretive considerations.

Trial; Setting	Positive PSMA Criterion	PSMA-Low or Discordant Disease Rule	Interpretive Consideration
VISION; post-ARPI, post-taxane mCRPC [3]	At least one metastatic lesion with uptake visually greater than liver; central eligibility read	PSMA-negative lesions excluded if a lymph node was ≥2.5 cm in short axis, a solid-organ lesion ≥ 1.0 cm in short axis, or a bone lesion had a soft-tissue component ≥ 1.0 cm in short axis; FDG PET not required	Broad visual threshold with exclusion of clinically significant target-negative disease
TheraP; post-docetaxel mCRPC [5,31]	SUV_max_ ≥ 20 at one disease site and >10 at all other measurable sites	FDG PET required; FDG-positive/PSMA-negative lesions excluded	High-intensity, dual-tracer enrichment selected a population with high PSMA uptake and no major FDG discordance
PSMAfore; taxane-naive mCRPC [4]	At least one PSMA-positive lesion using liver-referenced central visual assessment	Patients with qualifying exclusionary PSMA-negative lesions were ineligible; FDG PET was not required; the size-based exclusion rule is distinct from PSMAddition’s liver-metastasis rule	VISION-like selection moved earlier in mCRPC
ENZA-p; first-line high-risk mCRPC [6,34]	Primary report: SUV_max_ ≥ 15 at ≥1 site and ≥10 at all larger sites not affected by partial-volume effect	FDG findings were not used to determine imaging eligibility	Quantitative criteria supported adaptive treatment and imaging-biomarker analyses
UpFrontPSMA; de novo high-volume mHSPC [7]	SUV_max_ > 15 at one or more disease sites	FDG PET required; major FDG-positive/PSMA-negative discordance excluded	Selected a high-uptake, metabolically concordant early-disease population
PSMAddition; mHSPC [2,9,32]	Metastatic lesion(s) on conventional imaging and ≥1 tumour lesion with [^68^Ga]Ga-PSMA-11 uptake greater than normal liver; central eligibility read	FDG PET not required; excluded if all present liver metastases were PSMA-negative	1232/1420 patients screened by PSMA PET/CT (86.8%) were centrally positive; PSMA PET-only metastases were not represented

Abbreviations: ARPI, androgen receptor pathway inhibitor; FDG, fluorodeoxyglucose; mCRPC, metastatic castration-resistant prostate cancer; mHSPC, metastatic hormone-sensitive prostate cancer; PET, positron-emission tomography; PSMA, prostate-specific membrane antigen; SUV_max_, maximum standardised uptake value. ENZA-p thresholds here follow the primary efficacy report (≥15 and ≥10) [6]; the later biomarker methods describe ≥15 and >10 at other measurable sites [34]. Exact protocol definitions and lesion-size rules remain relevant. The table does not introduce additional clinical eligibility criteria. Reading arrangements refer to eligibility assessment, not endpoint review. Eligibility reading arrangements and lesion-size rules are summarised as described in the cited trial reports and should not be transferred between trials. The liver reference is tracer-dependent [33].

**Table 4:** Evidence map for the author-proposed five-domain framework.

Domain	Key Clinical Question	Supporting Evidence and Certainty Limits	Multidisciplinary Focus
Target	Is clinically important disease sufficiently PSMA-accessible?	Direct phase III eligibility evidence [2]; quantitative and heterogeneity biomarkers largely contextual or exploratory [31,34,35].	Trial/label eligibility, target-poor high-risk lesions, tracer and reading differences.
Disease	How urgent is additional control, and which alternatives are appropriate?	Direct randomised mHSPC efficacy [2,21,22,23,24,25,26,27,28]; volume and presentation inform prognosis, not a validated differential radioligand-benefit rule.	Burden, tempo, symptoms, anatomical risk, and chemotherapy fitness.
Patient	Do likely benefits justify toxicity and burden for this person?	Trial safety and patient-reported outcomes [2]; organ-assessment guidance largely derives from mCRPC [36,37]. Long-term mHSPC safety remains uncertain.	Marrow/renal reserve, comorbidity, life expectancy, values, preferences, and practical tolerance.
Sequence	How might treatment now affect later options?	PSMAddition crossover and immature PFS2 [2]; retreatment evidence is selected and non-randomised [45]. Optimal lifetime sequencing is unestablished.	Subsequent therapy, cumulative exposure, retreatment alternatives, and trials.
System	Can appropriate treatment be delivered equitably and on time?	Procedural guidance [36,37] and clinical implementation considerations; not evidence of biological treatment eligibility.	Supply, capacity, coordination, travel, caregiver support, and geographic/equitable access.

Evidence descriptions indicate source type, directness, and limitations; no formal certainty grading or consensus procedure was undertaken. The domains are considered together and do not define a score or additional eligibility restrictions. PFS2, second progression-free survival; other abbreviations are defined in the text.

## Data Availability

No new data were created or analyzed in this study. Data sharing is not applicable to this article.
